# Effects and Causes

**Published:** 1883-05

**Authors:** J. A. Robinson


### THE DENTAL REGISTER.

VOL. XXXVII.] MAY, 1883. [No. 5.

### COMMUNICATIONS.

### Effects and Causes.

BY J. A. ROBINSON, D.D.S.

[Read before the Michigan State Dental Society.]

The dentist, the physician, and, in fact, every person who deals in the phenomena of this life deals in effects, because he is in a world of effects. We see the effects: they meet us on every hand, and while the scientists, the philosophers, and the theologians have been these thousands of years trying to ascertain the cause of the various phenomena of life, the real cause is never obvious, and can never be known, because all causes are spiritual, and cannot be fully realized by a finite mind.

It is all very well to endeavor to divine the cause, and man is so constituted that he will be continually striving in that direction on account of the spiritual nature within him that is always leading him higher in attainments towards the limitless ocean of experiences that lie before him.

I am aware that some of you may think that everything pertaining to the human system can be decided by physiological laws, but you must not forget that all physiology does is to treat of the properties and functions of living beings as they appear to us in their several forms, and in their relation to their natural life, which is only an effect produced by some spiritual law that has never yet been understood. We can explain the laws of *effect* by certain hypotheses and investigations and the facts relating to them, but when we come to the laws of mind, and especially the laws of life and love and substance, it is a branch of science that treats of things spiritual and cannot be fully understood by the finite mind.

The recent discussions among dentists in regard to the etiology of the teeth; the acid theory as against the germ theory may after all be only a different phenomena of the same thing, like the endless discussions as to which was first, the hen or the egg. The convergence is the same as that among theologians in regard to divine sovereignty and man's free agency.

Each like a pillar rises to the sun,
And in celestial brightness blends in one.

The same difference exists among physicians in regard to pulmonary diseases; the germ theory has it advocates, and there are as many dissenters. This disceptation is productive of great good because it promotes investigation, and that is the real road to progress. Of the fifteen thousand dentists in the United States, not more than five hundred are interested in the cause of disease and of the theory of decay, while every one is interested in the cure.

In an abstract sense man can never comprehend what absolute truth is, or substance, or love, while they all converge towards one center which is life. Relatively he may; but, relative to what? If it be relative it must relate to something that is beyond the appearance—that is *real.* So we say the apparent thing is only the effect of some cause, and as all original causes are really beyond our comprehension they must be spiritual. Perhaps in the end we may find that it is all the various elements combined in a vitiated state that cause bacteria, and that the life of bacteria is an acid, as in vinegar when the animalcula are the more numerous the vinegar is the sharpest, and when the life of the vinegar is gone and it is dead, or destitute of the animalcula, it is no longer acetic acid but some dead and stagnant substance.

It is very hard to tell which particular passion has demoralized the man, but the man is degraded all the same, and it is a combination of passions as a rule that destroys him, and not any one particular thing.

Chloroform is the great remedy in neuralgia in nearly all its forms. After tooth extraction it is instantaneous when applied to the cavity from which the tooth is taken. So after a tooth has been filled and slight congestion of the periosteum or engorgement of the blood vessels results in elongation and soreness and inflammation that it seems unbearable, this remedy, applied to the free margin of the gum of the tooth that is affected, will in almost every case give immediate relief; as also when it is applied to the parts affected in general neuralgic pains it is a sure remedy. And yet why chloroform produces this effect upon the human system is not positively known. We know the effect and the fact, and it is not empyricism when it does not fail to do the same thing once in a hundred times. It takes but two things to make a good dentist, and these two things are: love for the work and ability to do it. So in tooth extraction it can hardly be said that these two faculties are brought into action; for, when a tooth is extracted it is *dead*, all love for it ceases, and all desire to make it better has passed away—and what gave it its life is unknowable.

It could hardly be understood, even by special inspiration, as claimed by Dr. Atkinson.

Inspiration is knowledge obtained through the spirit of love. What we get through the lower forms of our being, what we perceive through the senses, are only impressions; they belong to the external world, but what we see with the finer qualities of the mind, as love, charity, benevolence, justice, and those things that go to make up our christian civilization and lead us to obey the Golden Rule, are inspiration; they do not occupy time and space, nevertheless we know they exist, and we realize them by their manifestations as we realize the emotion of charity by carrying the basket to relieve those who are suffering. So, whenever Dr. Atkinson is bitter and sarcastic to his opponents and insinuates that they are imbeciles and idiots, he is dwelling in the impressions on the lower forms of his nature, and cannot claim to be an inspired man in such moments of vexation and disquietude. The most learned men in the profession have honest differences of opinion in regard to the cause of inflammation of the mucus surfaces of the mouth brought about by wearing rubber and celluloid plates for artificial dentures. Every dentist knows that inflammation exists, and while some contend that it is produced by the sulphuret of mercury contained in the plate, others state as positively that it is the heat generated by wearing vegetable plates.

We all know the effect, but who can tell us the cause? It is the effect we have to deal with, and all appliances to remedy the evil are a blessing to the race and correspond to the brakes on the train of cars to save human life and prevent our going to destruction.

Whatever is going to decay is substance changing form; but what substance is, man can never comprehend.

In this controversy in regard to bacteria and acid, the difference is not so much in point of fact as in theory. One mind sees the great universal of the world as a whole, while another mind beholds a sort of personality, in all things.

In the end every particle must have some sort of real personality, and, as in decayed teeth and those that are not decayed, when the substance is changing form to a sort of leathery appearance and has passed the dead line from the living tissue to decay, there is always a portion of substance that is alive which is only a short distance behind what is already dead that seems to be food for another life whether acid or bacteria, or both. It is like the inner life that we really have that can only be seen by its manifestations. Thoreau saw the leaves on the trees break forth into singing like the psalmest, and never looked upon an acorn but he saw in that acorn the sturdy oak, and never contemplated a single bud but he saw it swelling forth into the victory of life. So every dentist sees the same thing when he takes the heterogeneous gold and forms the concrete filling.

When we save teeth by filling, we are acting on the same principle *outside* that the creator does *inside*, when he is rejuvenating a plant or a flower, because we are engaged in a labor of love.

All things from the rock up to man are only different manifestations of the same life and only differ in degree.

The mineral is a form of life for the lowest use, the vegetables are forms of uses for the life of man, who is only a step higher and is the most perfect in life and form. The reason the human soul is inspired at the sight of the beautiful, is, because the beautiful is always true.

Mr. Emerson says, " Beauty is the form in which the intellect prefers to study the world, and whoever adorns his work will never tire of doing it." This is particularly true in our own profession. When a dentist sees a tooth that has been filled for twenty years and has stood the test of time, he is filled with the same feeling in kind that the soldier is in seeing the flag of his country or the trophies of war. They are symbols of victory after a hard fought battle that was successful. He is inflamed with courage that is a sort of guarantee of success in the future. The reason we are moved by eloquence or music, is because the forms of music and eloquence find a vibration within ourselves; so in filling teeth the *form* artistic or otherwise, like sculpture, holds us in admiration of the work till it is finished and we reproduce our ideal fillings in the perfect work we are doing. One great objection to the use of amalgams in filling teeth is, that it requires no effort, and where there is no effort there can be no progress. We grow strong in proportion to the effort we make in doing the work.

In labors of art the sterility of man's mind becomes fertile which otherwise would lie dormant, and as we progress we are often obliged to unlearn things that cost us great labor to acquire. A teachable and willing mind is essential to success.

The imagination—that spiritual part of us—through which we communicate and speak to the animal organism to make known our wants and desires is strengthened by cultivation as much as the bodily functions and muscular powers, and it is through these spiritual faculties that we make progress in dentistry as well as in music and painting.

Bigotry is the child of ignorance, and skepticism is the child of knowledge; they both lead to aristocracy of self, and are real enemies of progress, while the home of all true greatness is built on the road of humility. As a common calamity dissolves all differences and caste in society, so a common effort in art draws all true artists into intimate relations and real friendships. The reason we place such stress on filling teeth is because next to human life is human health, and the mind rises in proportion to the subject to be treated, and we are moved to exertion just in proportion as the subject is greater in our mind and understanding. It is the fuel that kindles the fires in the heart. In filling teeth and in welding gold to make perfect work the finer faculties of the mind are brought out and strengthened.

Dr. Ure, in his dictionary of the minerals and metals, says: "To weld is to press or beat together into intimate and permanent union as two pieces of iron when heated almost to fusion."

Webster adopts Ure's definition but adds that very few of the metals besides iron are capable of being welded. While the above is true in regard to metals when heated almost to a point of fusion, it is also true that certain other metals can be brought into intimate relation and tolerable permanent union under pressure without perceptible heat being applied during the process of welding. There can be no doubt but an infinitesimal amount of heat is produced by filling teeth by hand pressure, and that it is increased by the use of the mallet, which gives mallet work the preference as far as solidity is concerned in tooth filling.

It is now so many years since the welding properties of gold as applied to dentistry, that few, except the older members of the profession, have any practical knowledge of the skill required in its manipulation when non-cohesive gold was used, and the difficulties attendant on the preparation of the cavities and retaining the gold in position firmly against the walls while the several pieces, as they were introduced, until the final key of the mass, much after the manner of the keystone of the arch prevented the whole structure from falling to pieces. It would be impossible to enumerate the different treatments to which gold has been subjected to bring it to its most excellent form for dental purposes.

It is enough to say that it is no longer a matter of doubt or dispute that it, when cold or apparently cold can be made into any desired form, and of almost the same specific gravity as ingot gold. This end is attained by an annealing process. When the gold is annealed the surface is covered with crystals of irregular size, but mostly octahedral in form; these when brought into contact with one another and pressure applied with points sufficiently serated to force within and interlock the crystals and still not destroy or flatten the surface cause the gold, owing to its great ductility, to become so firmly united in its parts, that with care it can again without remelting be rolled into plate. With tin and alloys composed largely of tin, the same results can be attained, but the process is somewhat different. Although tin is one of the most difficult metals to crystalize, by a combination of metals crystalization takes place and remains intact until it can be fused into an ingot and then cut with a machine into very fine shreds or fibres which being interlocked can again be welded by the use of similar instruments as those used to condense the gold into a mass of almost any shape or form. The crystals of gold and crystals of tin in the preparation of the new fibrous and metalic filling combined with platinum make a new metal for filling teeth. Platinum is ductile but non-expansive as a metal, but when combined with tin which is also a brittle metal and non-expansive, it seems more expansive than gold, and when cut into minute fibres, the crystals will coruscate and sparkle in the sunlight so they are preceptible with the naked eye.

This metalic compound, being plexiform, welds to gold almost as readily as gold welds to itself when the gold is soft and annealed so the surface is crystalized.

In cervical walls (say what we will) gold fillings are often failures, and this new metalic filling bears the same relation to filling teeth, that the grouting does to the superstructure of the building, and every builder understands that grouting is better for the sub-structure in leaky and miry places than even the solid granite. It has long been acknowledged by some of our best operators that tin, as a filling, has less conductive properties than gold, and many dentists assert that it possesses antiseptic qualities that arrest decay in poorly calcified teeth, but tin-foil recoils under pressure either by the hand or the mallet, while the fibrous metalic filling is absolutely without recoil, and experience proves that it changes color less in the fluids of the mouth than tin and takes a polish second only to gold. It is equally a non-conductor, and possesses all the therapeutic properties of tin-foil and is much more easily manipulated and adheres more closely to the walls of the tooth.

So when the substructure of the filling is made of the new material, and the gold welded to the new metal, it bears the same relation for service that the steel facing does to the hammer that is made of iron, and the combination makes the perfect instrument for service, as the combination of the two metals makes also the perfect filling. Fillings made in this way are not open to the objections of the plastics that wash, or to the amalgams that fail in almost every instance.

Most dentists know that in preparing gold for dental purposes, it is beaten between gold beater's skin in book form, and in the process the crystals are flattened until it is no longer cohesive, but when it is annealed or heated to about 400 degrees the crystals assume their wonted form as much as possible. The why crystalization takes place, or the why the crystals of gold assume their original form at 400 degrees of heat may never be known any more than why the amœba divides itself that it may reproduce itself, except that love and heat and life are the same thing called by different names; they are the effects we perceive of the unknown cause. Of this we may be sure, that all life is the same life reproducing itself continually, and that anything incompatible with the highest love is not life or truth; and as light is the form or expression of heat so truth is the form of love, and love is the form or expression of thought, and all are different manifestations of life, and this form of reasoning is the only satisfactory evidence we have of the endless life or immortality. The apparent cause of inflammation of the mucus surface of the mouth by wearing rubber plates is because there is no bond of union or affection between the surface of the rubber and the mucus surface of the mouth, but rubber has too many good qualities to be thrown away altogether, and the real cause of this incompatibility may never be fully known.

The various theories in regard to the failures of amalgam fillings are as much in dispute as those set forth in the beginning of this paper. Whether it is electro chemical fluids that act upon the tooth bone that cause the decay, or it is contraction by crystalization is still a matter of dispute? Is it no consequence whether the contraction of the amalgam admits the fluids of the mouth to dissolve the dentine so long as the same effects are produced by either or both the causes combined—for, as I said before, it is the effects more than the causes that we are particularly interested in. In learning, and what we have learned, the one is what is behind, and the other is what is before us. It is like hurrying and being hurried.

The duties of the dentist are two fold. His calling is as sacred as that of the physician or the moralist. He has a divine nature under his charge. He must be of serious mind and well informed that he may be fit to serve and teach others. There still exists great predjudice in the minds of many against advertising, but the only great crime I know about advertising is to advertise "teeth extracted without pain," or promising to do what we are unable to perform. Extracting teeth without pain is a temptation to destroy a part of the divine architecture, but associations like this and schools and colleges and clinics are to spread the light, and there is no way that it can be done so effectually as by the printing press.

If it is for the interest of the dentist to be well informed; it is for the interest of his patrons to be well informed in regard to all that pertains to the health and the preservation of their teeth. Who are the persons who give us the least trouble after service has been performed? Are they not those who are the best informed on the subject themselves? People prize everything in this life in proportion to what it costs them, and those who know most about the teeth are those who take the best care of them, and when they need the services of a dentist they are the first to attend to their duty, and, of course, will be our best patrons and will pay the most liberally. Suppose every person in the country was as well informed as those I see before me, would a charlatan in dentistry be employed? It is not because the people are not well informed on this subject that so many inefficient operators are abroad in the community. I do not mean the low catch-penny cheat in advertising that will deceive, but a broad general diffusion of dental knowledge that is full of love and light and truth that will save the race, and given under your own signature. The whole world is fast converging toward the freedom of the race, and professions will be the last to yield and let go their prerogatives and self-acquired privileges.

This convergance can be traced through all the paths in medicine, through all the isms of religion, through all governments towards republican institutions. It is removing all incongruities in society in general as we progress in education, and it is tending towards the unfolding of the cause of all causes that bind us to and make us part of the Universal Father of all.

Let us not forget that in the diffusion of knowledge we *must* grow. In giving information "man is twice blessed." When we begin to teach others we begin to learn ourselves. "It blesses him who gives and him who takes;" and while in former times, as among uncivilized races, secrecy was necessary to their preservation and very existence, the light of to-day should dispel all darkness that has hung like a pall over the past. If in this paper I have been dull and indulged in platitudes, and things that seem to be without meaning to some of you, you will not forget that truth is never a platitude to the honest soul; the Lord's Prayer is never a platitude to the devout soul filled with emotion; the multiplication table is not a platitude to the mathematician, for it is the key to all the higher mathematics. The science of dentistry, based on the principles I have suggested, can never be a platitude to the dentist, who, in his labor of love, has invested all his individuality in his profession, and in his desire to go on to still higher and loftier achievements.

